# A spectral analysis of stem bark for boreal and temperate tree species

**DOI:** 10.1002/ece3.8718

**Published:** 2022-03-16

**Authors:** Jussi Juola, Aarne Hovi, Miina Rautiainen

**Affiliations:** ^1^ Department of Built Environment School of Engineering Aalto University Aalto Finland; ^2^ Department of Electronics and Nanoengineering School of Electrical Engineering Aalto University Aalto Finland

**Keywords:** field measurement, forest, hyperspectral, imaging spectroscopy, reflectance

## Abstract

The woody material of forest canopies has a significant effect on the total forest reflectance and on the interpretation of remotely sensed data, yet research on the spectral properties of bark has been limited. We developed a novel measurement setup for acquiring stem bark reflectance spectra in field conditions, using a mobile hyperspectral camera. The setup was used for stem bark reflectance measurements of ten boreal and temperate tree species in the visible (VIS) to near‐infrared (NIR) (400–1000 nm) wavelength region. Twenty trees of each species were measured, constituting a total of 200 hyperspectral reflectance images. The mean bark spectra of species were similar in the VIS region, and the interspecific variation was largest in the NIR region. The intraspecific variation of bark spectra was high for all studied species from the VIS to the NIR region. The spectral similarity of our study species did not correspond to the general phylogenetic lineages. The hyperspectral reflectance images revealed that the distributions of per‐pixel reflectance values within images were species‐specific. The spectral library collected in this study contributes toward building a comprehensive understanding of the spectral diversity of forests needed not only in remote sensing applications but also in, for example, biodiversity or land surface modeling studies.

## Graphical abstract

The woody material of forest canopies has a significant effect on the total forest reflectance and on the interpretation of remotely sensed data, yet research on the spectral properties of bark has been limited. In this paper, we pioneered a new measurement method in quantifying intra‐ and interspecific differences in tree bark spectra of ten common boreal and temperate tree species. We also examined if such spectral information is linked to the taxonomic relatedness of the studied tree species.

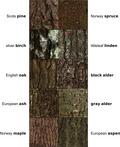

## 1 INTRODUCTION

The optical properties of leaves, understory, and woody tree structures have been studied using in situ and lab measurements (e.g., Gates et al., 1965; Miller et al., 1997; Williams, 1991), but there remains an imbalance between the amount of spectroscopic information available on the different forest components. This gap is important to fill because the different elements together constitute the total reflectance of forest canopies, albeit with varying weight. Research on the spectral properties of woody structures of trees (i.e., stem and branch bark) has been sparse, as reviewed by Rautiainen et al. (2018). Yet, woody material can contribute to as much as 5–35% of the total plant area in a forest (Gower et al., 1999). As such, woody material has a significant effect on the total forest reflectance and on the interpretation of remote sensing data (Hall, Huemmrich, Goetz et al., 1992).

The spectral signatures of woody tree structures are highly variable in reflectance. The variability is due to species‐specific differences in optical properties, which are driven by biochemical and structural traits of bark material, and moisture (Roberts et al., 2004). In general, bark spectra can be described as “soil‐like,” in that they increase monotonously as a function of wavelength (Rautiainen et al., 2018). However, there are considerable differences in bark spectra depending on tree species (Hadlich et al., 2018), bark moisture content (Elvidge, 1990), or position of the woody material in the canopy (Juola et al., 2020). The majority of previous studies that analyzed the variability of bark spectra were based on measurements of detached pieces of bark, which were conducted in the laboratory with a spectrometer and an integrating sphere (Asner, 1998; Campbell & Borden, 2005; Forsström et al., 2021; Roberts et al., 2004; Williams, 1991), whereas less research has been conducted outdoors for bark on living trees (Girma et al., 2013; Hadlich et al., 2018). Better understanding of bark optical properties in their natural state and environment would provide valuable and complementary knowledge to studies conducted in the laboratory with destructive sampling that require the cutting of branches or felling of trees. In addition, Hovi et al. (2021), Lang et al. (2002), and Spencer and Rock (1999) have published open‐access data of bark spectra that can be used as reference data in remote sensing. Very recently, there has been also research on the spectro‐directional behavior of stem bark of trees (Juola et al., 2020). However, to our knowledge, there has not been a study that has collected systematically a spectral library of stem bark of boreal and temperate tree species of Europe and analyzed the intra‐ and interspecific variations of the spectra. Spectral libraries with species‐specific spectra could be utilized, for example, as input in forest reflectance models.

Understanding the intra‐ and interspecific variation of plant spectra is also important for species identification, and for studying taxonomic relatedness. For example, classification of tree species based on stem bark spectra was demonstrated by Hadlich et al. (2018), but there could also be potential in assessing taxonomic diversity of a vegetation community through its spectral variation. Overall, investigating the links between phylogeny, functional traits, and optical properties of plant species has seen increasing interest in biodiversity research (e.g., Asner & Martin, 2016). However, to date, the majority of studies interested in phylogenetic relatedness and taxonomic diversity have analyzed leaf optical properties (Asner & Martin, 2011; Asner et al., 2009, 2011, 2014; Frye et al., 2021; McManus et al., 2016; Meireles et al., 2020; Schweiger et al., 2018). Woody structures, such as bark, have not been utilized as widely (Lang et al., 2017), and therefore it remains unknown if taxonomic relatedness of species could be determined from bark spectra.

In this study, our goal was to collect and analyze a spectral library of bark of European tree species. First, we constructed a measurement setup for a mobile hyperspectral camera that is applicable in demanding forest conditions. Then, we used the setup to collect a spectral library of stem bark spectra and respond to the following research questions:

1. What are the intra‐ and interspecific variations of bark spectra between 415 and 925 nm?
2. Is it possible to detect taxonomic relatedness of our study species based on reflectance spectra of bark?
3. How does reflectance of bark samples vary within hyperspectral images?

## 2 MATERIALS AND METHODS

### 2.1 Study site and stem bark samples

**FIGURE 1:**
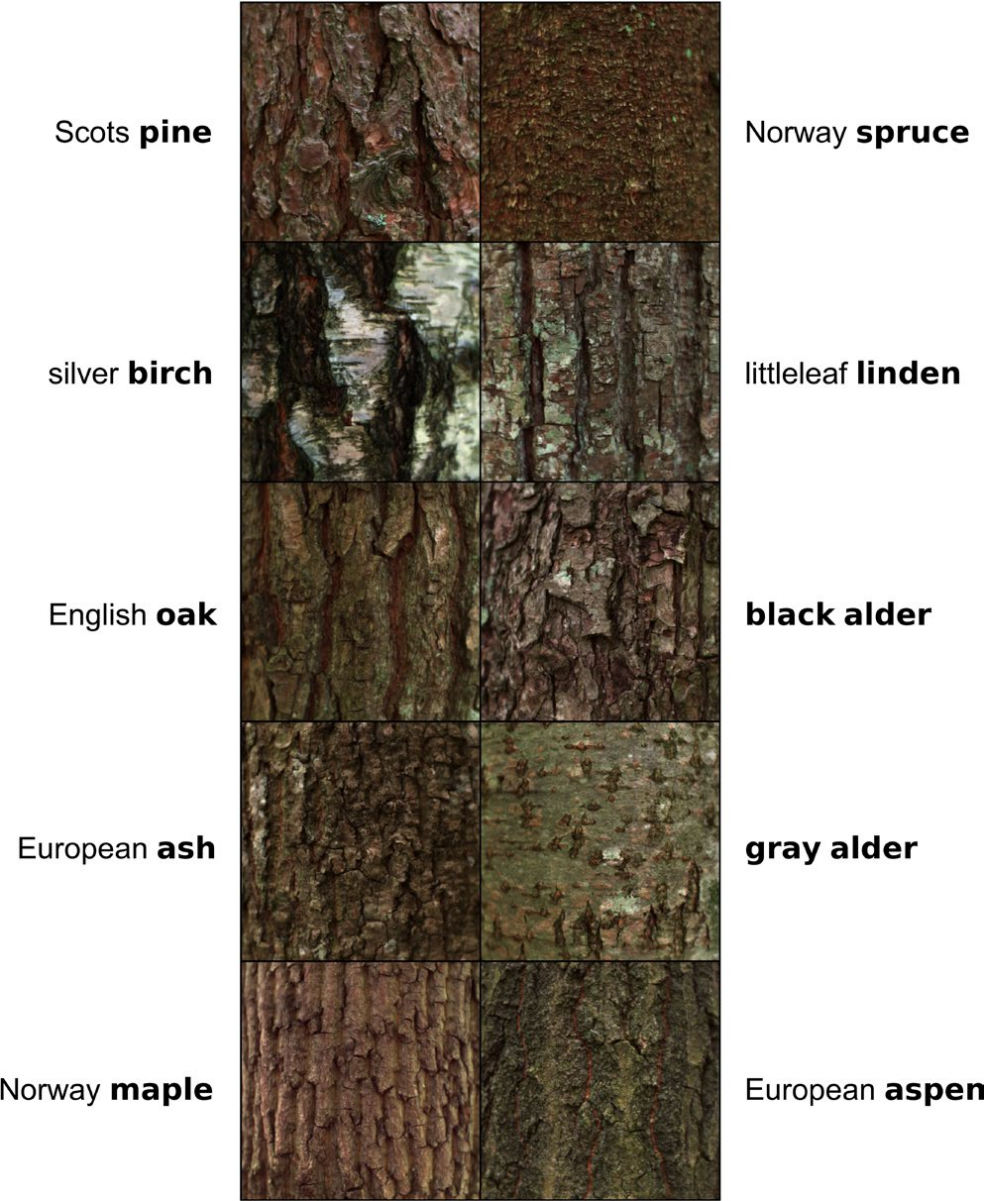
Visual examples showing a subset of tree stem bark samples of the study species (red‐green‐blue image composites derived from original hyperspectral reflectance images)

We measured in situ reflectance spectra of stem bark of ten common tree species found in boreal and temperate forests of Europe (Table 1). The main study areas were located in Finland within the Capital Region (approximate coordinates: Viikki Arboretum 60°13′13″N 25°00′25″E, Helsinki Central Park 60°15′36″N 24°55′19″E, and Espoo Central Park 60°11′13″N 24°42′25″E), and in Estonia in proximity to the Järvselja Training and Experimental Forestry District (approximate coordinates: 58°17′13″N 27°18′50″E). We measured 20 trees per species, accounting to a total of 200 unique stem bark samples. One sample refers to a hyperspectral reflectance image taken of a single tree stem (Figure 1). The measurement campaign was conducted from May to August 2020.

**TABLE 1:** List of measured tree species, families, and structural characteristics of the measured trees and forest stands they were located in. Bolded names are used throughout this study. Number of samples was 20 per species

Species		Family	Tree diameter at breast height [cm]			Tree height [m]			Stand basal area [m^2^/ha]
Scientific	Common		Min.	Max.	Median	Min.	Max.	Median	Min.–Max.
*Pinus sylvestris* L.	Scots **pine**	Pinaceae	16	47	28	10	30	18	12–44
*Picea abies* (L.) Karst	Norway **spruce**	Pinaceae	15	47	26	13	31	24	22–48
*Betula pendula* Roth	silver **birch**	Betulaceae	15	36	25	17	29	22	8–36
*Alnus incana* (l.) Moench	**gray alder**	Betulaceae	15	26	19	12	26	20	12–48
*Alnus glutinosa* (L.) Gaertn.	**black alder**	Betulaceae	18	36	25	20	30	24	18–52
*Quercus robur* L.	English **oak**	Fagaceae	21	40	29	19	28	25	30–54
*Populus tremula* L.	European **aspen**	Salicaceae	20	44	29	17	31	25	22–42
*Fraxinus excelsior* L.	European **ash**	Oleaceae	15	47	30	10	37	24	20–52
*Acer platanoides* L.	Norway **maple**	Sapindaceae	19	42	26	16	27	21	26–48
*Tilia cordata* Mill.	littleleaf **linden**	Malvaceae	18	48	31	19	33	28	36–72

Minimum diameter at breast height (DBH) for the measured trees was 15 cm. This was to include trees with commercial value, but also to ensure uniform measurement and analysis protocol for all trees: the image size in our measurement setup was 11.1 × 11.1 cm, and therefore measuring small trees would have required either bringing the camera closer to the tree (i.e., reducing the pixel size), or using only a limited area of the image. Thus, the measured stands were selected so that a sufficient fraction of trees exceeded the minimum DBH. The site fertility varied, but all stands were on forest land and were tree‐covered (basal area >8 m^2^/ha), that is, measurements were not performed for solitary trees in parks or on, for example, extremely barren rocks or peatland. We measured tree height and DBH of every sampled tree, together with the basal area of each of the surrounding stands (Table 1).

### 2.2 Sample selection

The same measurement protocol was followed throughout the campaign to maintain repeatability and to ensure as objective and randomized sampling as possible. In each forest stand, an imaginary North‐to‐South line was drawn with a hand‐bearing compass. This North‐to‐South line denoted the path from which every third tree was selected, and a hyperspectral image was taken from the northern side of the tree trunk at breast height (1.3 m). The northern side of the tree stem was measured to support diffuse and stable measurement conditions and to avoid possible sunflecks, sunpatches, and shadows (i.e., effects of sunlight penetration through the canopy) (Smith et al., 1989). Each drawn measurement line was allowed an approximately 2 m perpendicular buffer to the sides, which helped to select sample trees even in more sparse forest stands. To ensure variation of forest stands and individual tree ages, we measured a minimum of two separate stands per species and a maximum of ten trees per stand.

In addition to the minimum DBH requirement, if the tree stem had qualities at breast height that made measurements impractical or useless (e.g., damaged stem, large growth of epiphytal vegetation, or abnormal angle of stem), the next tree in the compass line was measured instead. A visual limit of 10% for epiphytal vegetation (e.g., lichens or fungi) and other nonstem material (e.g., branches) was set to ensure as pure bark samples as possible, while allowing small amounts of naturally occurring features to remain. The physical state of the bark in the sample area was not altered.

### 2.3 Mounting setup

**FIGURE 2:**
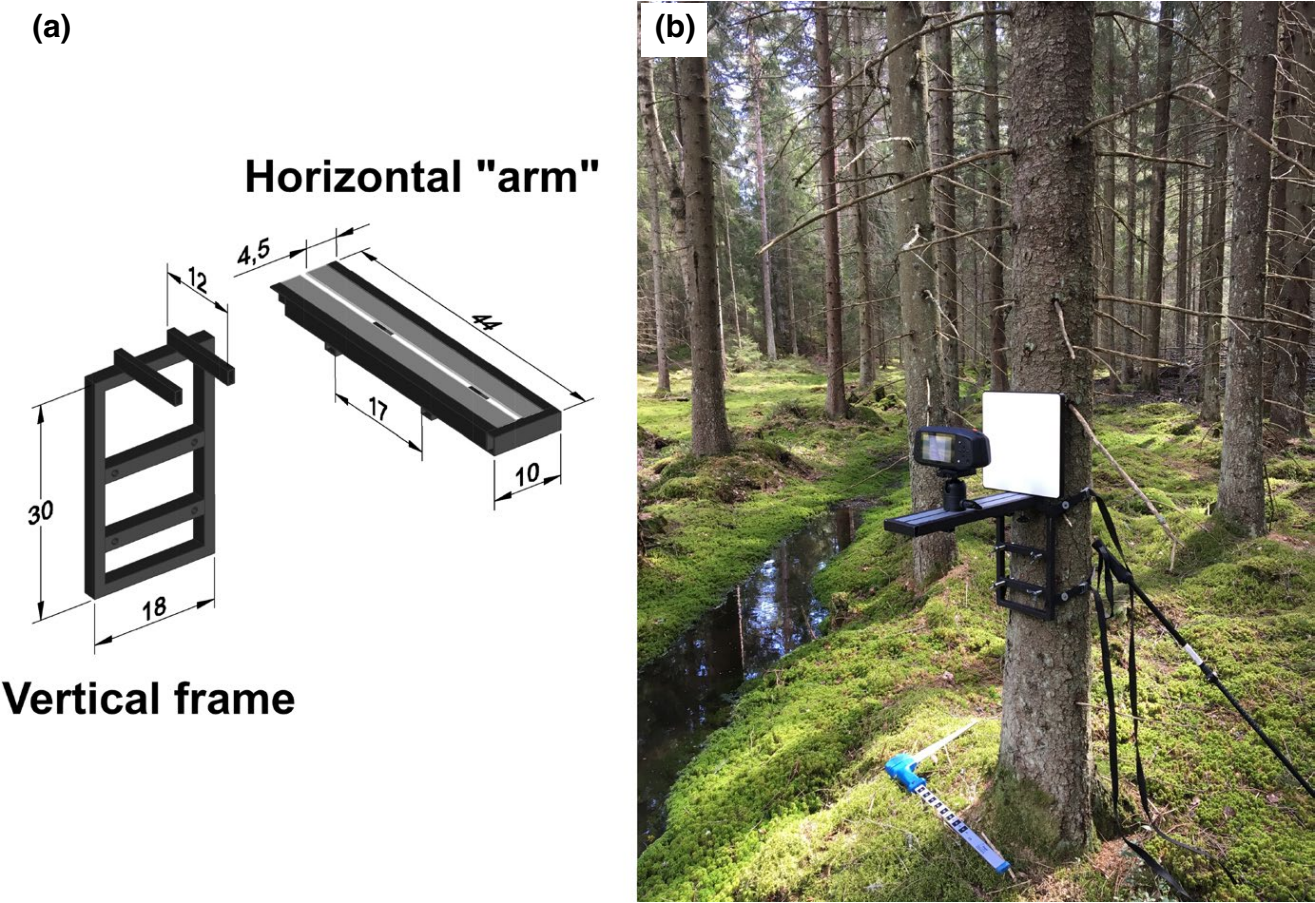
(a) Sketch of the measurement setup used for mobile hyperspectral imaging of tree stems (dimensions in centimeters), and (b) example of the measurement setup when attached to a tree stem in the field

We designed and constructed a novel camera mounting setup for tree trunks, made specifically for field measurement conditions (Figure 2). Common challenges for measuring tree stem bark in the past have included unstable measurement geometry (e.g., spectrometer or white reference panel held approximately in place by hand), labor‐intensive protocol (e.g., measurements have required two operators), use of impractical measurement accessories (e.g., tripods), and fixed sensor alignment (e.g., inflexible measurement distance or sensor angle).

The frame for the entire setup was constructed of steel piping that were measured, fitted, and welded to produce two separate yet interlocking pieces (Figure 2a). The horizontal “arm” with a fitted aluminum railing allowed the camera to be mounted on a standard ball head mount that can be locked to a custom distance (Figure 2). In addition, the railing made it possible to attach a white reference panel firmly onto the setup (Figure 2b). The horizontal “arm” was built with two receiving ends in the piping, which could be pushed onto the horizontal bars welded on the vertical frame (Figure 2a). The two interlocking pieces were tightened together with two screws below the horizontal “arm.” The system was built this way to be able to disassemble the framing into a backpack that could be carried even in field measurement conditions. The two separate pieces were fitted to be 90° relative to one another, and the ball head mount had a bubble level that was used to verify that the camera was perpendicular to the tree stem during measurements. The entire frame was secured to the tree stem with two drivebelts and four stabilizing bolts (Figure 2b). The belts and stabilizing bolts secured the frame from rotating around the stem at a set height and allowed the user to level the entire system to be perpendicular to the tree stem (Figure 2b).

### 2.4 Spectral measurements

We measured hemispherical‐directional reflectance factors (HDRF) (Schaepman‐Strub et al., 2006) of stem bark samples. The measurements were made with a Specim IQ (Specim, Spectral Imaging Ltd., Oulu, Finland, serial number: 190‐1100152) mobile hyperspectral camera that was attached to the mounting setup (Figure 2b). The spectral range of the hyperspectral camera is 400–1000 nm, which covers the regions of visible (VIS, 400–700 nm) to near‐infrared (NIR, 700–1000 nm). The spectral resolution (full‐width‐at‐half‐maximum) of the camera is 7 nm and the total number of bands recorded is 204. Field‐of‐view of the camera is 31° × 31° and the dimensions for the image are 512 × 512 pixels. The hyperspectral image (i.e., data cube) with dimensions of 512 × 512 pixels by 204 bands is constructed by the camera with a pushbroom line scanner with a built‐in motor. Further technical details of the camera have been published by Behmann et al. (2018). The image acquisition distance of the setup was 20 cm, which provided image size of approximately 11.1 × 11.1 cm on the tree stem surface.

All measurements were made under natural illumination conditions and the images were taken in perpendicular view‐angle to the tree stem (Figure 2). To ensure high‐quality data, we measured during cloudless days or days with standard overcast sky, and because the measurements were made on the northern side of the stem, the illumination on the sample was always diffuse. The solar zenith angle during the measurements varied between 35 and 65°. The white reference was a calibrated 25.4 × 25.4 cm Spectralon^®^ (Labsphere Inc., serial number: 01EC‐8777) panel that has nominal reflectance of 99%. We collected a white reference image and a sample image for every measured tree, which enabled HDRF(*λ*) to be calculated for each pixel with the following equation:

(1) $$\mathrm{HDRF}\left(\lambda \right)=\frac{{\mathrm{DN}}_{s}\left(\lambda \right)‐{\mathrm{DN}}_{s\_\mathrm{dc}}\left(\lambda \right)}{{\mathrm{DN}}_{\mathrm{wr}}\left(\lambda \right)‐{\mathrm{DN}}_{\mathrm{wr}\_\mathrm{dc}}\left(\lambda \right)}\times \frac{t_{\mathrm{wr}}}{t_{s}}\times R_{\mathrm{wr}}\left(\lambda \right)$$

where,

*λ* is the wavelength of radiation [nm],

DN_s_(*λ*) is the digital number from the sample image,

DN_s_dc_(*λ*) is the dark current digital number from the sample image,

DN_wr_ is (*λ*) the digital number from the white reference image,

DN_wr_dc_(*λ*) is the dark current digital number from the white reference image,

*t*_s_ is the integration time of used for the sample image [ms],

*t*_wr_ is the integration time of used for the white reference image [ms],

*R*_wr_(*λ*) is the calibrated reflectance value for the white reference panel.

Integration times were selected manually, both for the white reference and the sample image, which is taken into consideration in Equation 1 (*t*_wr_ and *t*_s_). The equation (Equation 1) was formulated according to the device manufacturer's guidelines. Acceptable signal strength was ensured by monitoring intensity histograms computed by the camera, and data were checked to not include any overexposed (signal saturated) pixels. Entire processed HDRF images were used for data analysis (i.e., HDRF was calculated using all 512 × 512 pixels and 204 bands). Consequently, data sizes were equal for all species throughout the analysis. After calculating HDRF with Equation 1, the spectral range was clipped to include only wavelengths between 415 and 925 nm. This was done due to a low signal‐to‐noise ratio between 400 and 415 nm and an instability of the signal between 925 and 1000 nm (Behmann et al., 2018). Remaining spectra did not require further smoothing with any filter.

### 2.5 Spectral analysis

A mean spectral signature was calculated for each sample by averaging the HDRF values over all 512 × 512 pixels per wavelength. To calculate spectral variation between samples, we then further processed the samples’ mean spectral signatures into wavelength‐specific standard deviation and coefficient of variation of HDRF for each species. To quantify the intra‐ and interspecific variation in HDRF spectra, we used least squares estimation to fit a linear model (stats, R Core Team, 2019), in which HDRF was the response variable and tree species the (categorical) explanatory variable, and computed the coefficient of determination (*R* ^2^).

Multivariate analysis was conducted through two well‐established linear dimensionality reduction methods, principal component analysis (PCA) (Hotelling, 1933; Pearson, 1901) and linear discriminant analysis (LDA) (sometimes referred to as canonical discriminant analysis) (Fisher, 1936). Both analysis methods were applied separately on the full dataset having the same number of samples and features (i.e., wavelengths). The methods were used to reveal possible interspecific similarities in our study species based on stem bark spectra and assess the intrinsic dimensionality of the data. For clarity, only the dimensionality reduction property of the LDA was applied to the data. This means that it was utilized as a linear transformation technique to project a feature space into a smaller subspace, and not used as a classifier. Both algorithms were implemented with a widely used and published library called scikit‐learn (version 0.24.1) coded in Python (Pedregosa et al., 2011).

PCA seeks to transform the original input data (e.g., the hyperspectral data in this study) from a high‐dimensional space to a lower‐dimensional space through linear combinations of the input features, while maximizing the variation found in the dataset (Jolliffe, 2002; Pearson, 1901). PCA can be described as an unsupervised method, meaning that the known class labels (e.g., tree species) are not considered, and the method relies purely on the input data itself. The data are transformed into a new coordinate system, where the new set of orthogonal and uncorrelated features are called principal components (hereafter PCs) that maximize the percentage of variance retained from the input data in ascending order (i.e., PC1 holds maximum percentage of the total variance, PC2 holds less than PC1 but more than PC3, and so forth). It is common, but not always the case, that the first few PCs retain most of the variance found in the input data, which can be used to assess redundancy in the hyperspectral data and possibly improve its interpretation. The number of PCs selected for further analysis was chosen according to the number of PCs that retained 95% or more of the total variance, or minimum of three PCs. The input data to our PCA (as well as to the LDA) were all in the same reflectance units (HDRF). No additional scaling was used.

LDA is a dimensionality reduction method similar to PCA. The LDA output features, called linear discriminants (hereafter LDs), are linear combinations of the input variables that represent the most discriminative directions obtained from the new projection. Rather than maximizing the variation found in the input data, like in PCA, the LDA seeks to maximize the between‐class variance among the classes in the dataset (Hastie et al., 2009; Zhao & Maclean, 2000). LDA is considered as a supervised dimensionality reduction method because known class labels are required and considered in the algorithm. This means that the method relies on the input data and given sample‐specific class labels. This makes LDA well suited for separating possibly spectrally overlapping tree species using the high‐dimensional hyperspectral measurements of their bark. The output features (or LDs) of LDA are restricted to be number of classes minus one. Consequently, for our data, all nine LDs were used for further analysis.

From the outputs of PCA and LDA (taking curse of dimensionality (Bellman, 1957) into consideration), we gained input data for a hierarchical clustering analysis, which was used to group the spectral samples based on distances computed between them. The goal of this analysis was to investigate how spectral similarities (i.e., distances between clusters revealed by PCA or LDA) correspond with the taxonomic relatedness of our study species. We performed an agglomerative clustering, where the grouping method was bottom‐up, that is, the algorithm begins at the bottom where each sample starts in its own cluster. From there, the clustering continues by combining two closest clusters into one. The two single clusters are then removed from the pool and the new combined cluster is added. Each iteration of the grouping maintains and updates a distance matrix between the new and remaining clusters within the pool. For the agglomerative clustering, we used the Ward method with Euclidean distance metric from the SciPy library (version 1.6.1) coded in Python (Müllner, 2011). A dendrogram was formed to illustrate the results.

Finally, within‐sample variation of reflectance was examined through a histogram analysis. Histograms were computed with 200 bins for four representative wavelengths: (1) blue (493 nm), (2) green (560 nm), (3) red (664 nm), and (4) NIR (866 nm). The within‐sample histograms were averaged over all samples belonging to one species to produce mean histograms for each species and for each wavelength.

## 3 RESULTS

### 3.1 Intra‐ and interspecific variation

**FIGURE 3:**
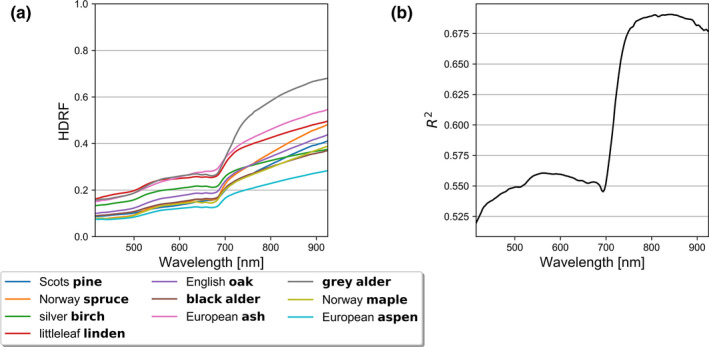
(a) Species‐specific mean reflectance (hemispherical‐directional reflectance factor, HDRF) spectra for ten different tree species, and (b) coefficient of determination (*R* ^2^) of linear model that explained variation of reflectance in visible to near‐infrared wavelength regions (415–925 nm), using tree species as a categorical explanatory variable

Interspecific similarities (i.e., nearly overlapping mean reflectance spectra) were found prominently in VIS, while distinct differences between tree species were found in NIR (Figure 3a). Visual inspection of the overlaps revealed that the mean reflectance spectra seemed to form two general groups in VIS. The first group with higher reflectance in VIS contained ash, gray alder, and linden, whereas the second group with lower reflectance in VIS was formed by aspen, black alder, maple, oak, pine, and spruce (Figure 3a). Birch fell between the two groups in VIS (Figure 3a). Between the VIS and NIR regions, the bark spectra exhibited the red‐edge effect (i.e., there was steeper slope in the otherwise monotonically increasing reflectance with wavelength) (Figure 3a). The largest interspecific variation was observable in the NIR wavelengths, where the separability between tree species was more pronounced (Figure 3a). Gray alder had the highest reflectance (up to 0.68) and aspen had the lowest reflectance (up to 0.28) within the NIR region (Figure 3a). According to the fitted linear model, tree species explained the variation of spectra most in NIR (Figure 3b). In summary, the measured tree species had similarly shaped mean spectra that varied in the level of reflectance (VIS to NIR). This was reaffirmed by an additional finding that reflectance values at individual wavelengths were highly correlated with each other within VIS and NIR regions, but reflectance at NIR wavelengths were not as strongly correlated with reflectance at VIS wavelengths.

**FIGURE 4:**
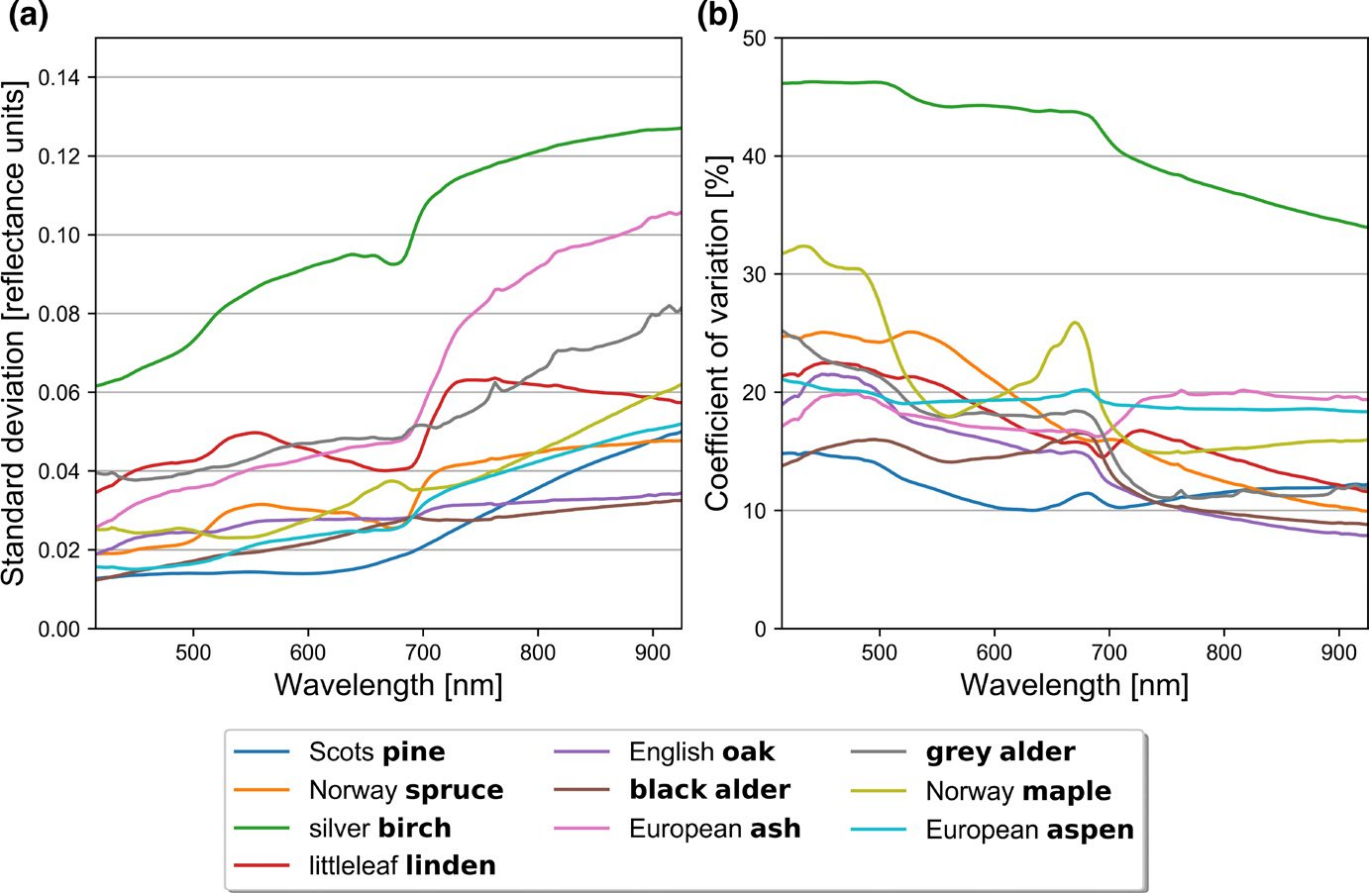
Interspecific (a) standard deviation and (b) coefficient of variation of tree stem bark reflectance samples from visible to near‐infrared wavelength regions (415–925 nm)

Intraspecific standard deviations were generally higher in NIR than in VIS (i.e., standard deviation increased from VIS to NIR as a function of wavelength) (Figure 4a). However, the coefficients of variation decreased from VIS to NIR (Figure 4b). More specifically, aspen, black alder, maple, oak, pine, and spruce had very similar standard deviations, which all varied between 0.01 and 0.06 in VIS to NIR, respectively. Birch stood out with standard deviation varying between 0.06 and 0.13, and coefficient of variation between 34% and 46% (Figure 4a,b). In addition to birch, ash, gray alder, and linden had slightly higher standard deviation in VIS (0.03 to 0.05) and in NIR, than rest of the measured tree species (Figure 4a). However, the coefficients of variation were relatively similar for all species except birch, varying between 8% and 32% in VIS–NIR (Figure 4b). The high standard deviation for birch was expected because the birch bark was mostly white with sporadic black spots (Figure 1). Finally, we examined a normal probability plot (a quantile‐quantile plot) for each tree species per wavelength, and birch was the only tree species in which the reflectance values were not normally distributed.

### 3.2 Multivariate analysis

#### 3.2.1 Principal component analysis

**FIGURE 5:**
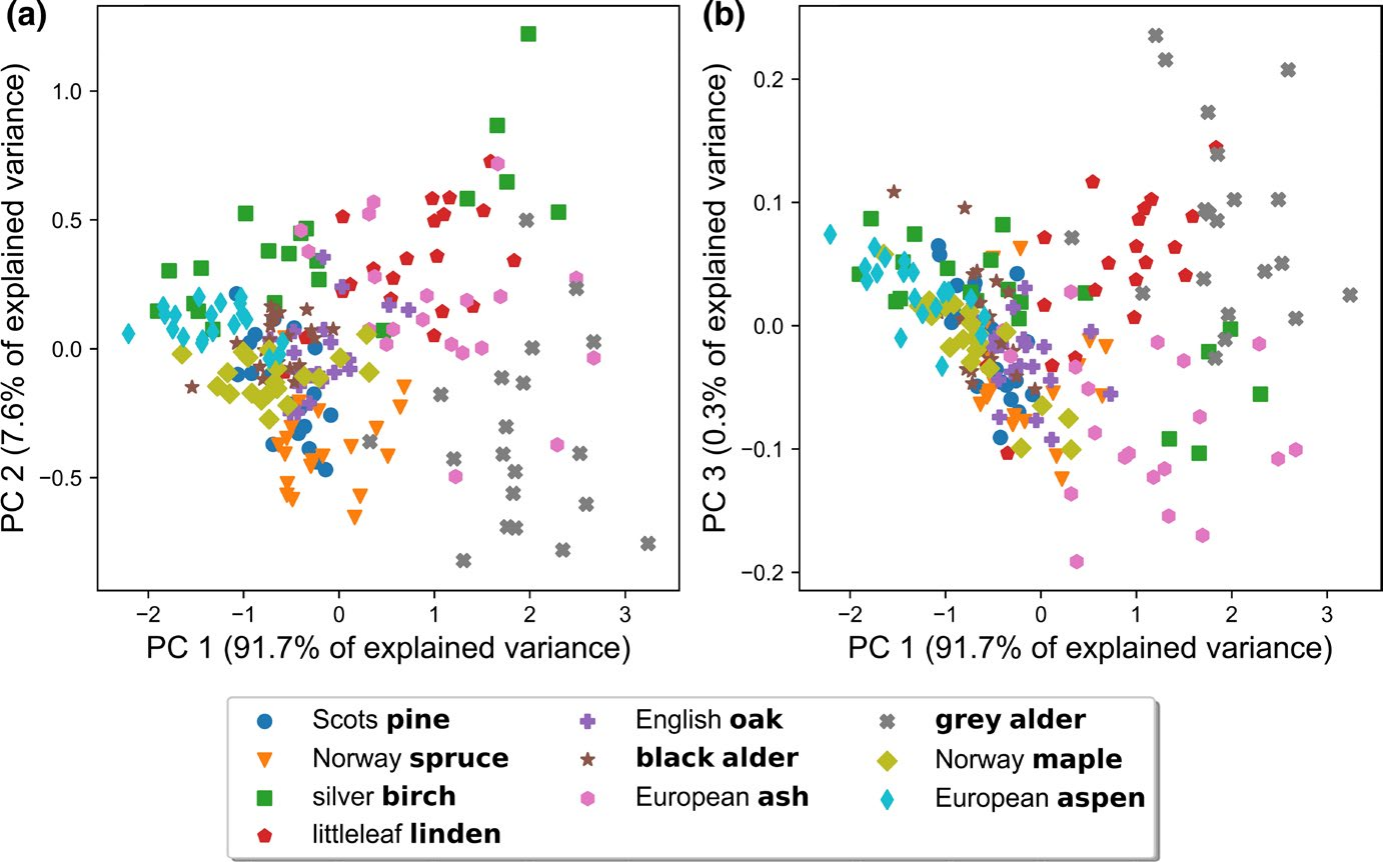
Two‐dimensional projections of bark reflectance samples into principal components (a) one and two (PC1 and PC2), and (b) one and three (PC1 and PC3) of the PCA. Axis labels show the percentage of explained variance by each component used

**FIGURE A1:**
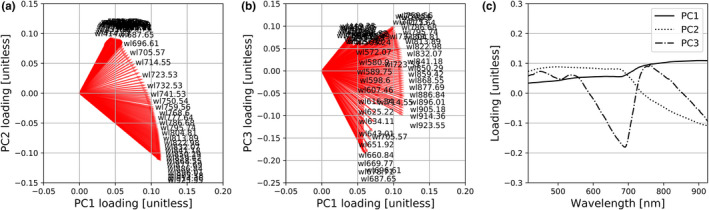
The loadings for the principal components (a) one and two as vectors (PC1 vs. PC3), (b) one and three as vectors (PC1 vs. PC3), and (c) one, two, and three (PC1–PC3, respectively) plotted against wavelengths. The red lines in (a) and (b) are the vectors and the black text with prefix “wl” at the end of each vector gives the wavelength it is connected to. Due to the correlation between the wavelengths, only every third label is shown in (a) and (b) to minimize overlapping of text

The first three PCs (PC1–3) of PCA explained 91.7%, 7.6%, and 0.3% of variance in the spectra, respectively. As such, PC1–3 were enough to capture 99.6% of the variance from the data. Plotting PC1 against PC2 exhibited several sparse species clusters when the tree species were viewed separately (e.g., aspen, black alder, maple, oak, pine, and spruce), but the results mainly highlighted the large intraspecific variation if all species and samples were viewed in the same space simultaneously (Figure 5a). Gray alder was slightly separable (with few samples of ash mixed in). Selecting a two‐dimensional space comprising PC1 against PC3 did not reveal additional information compared to the prior plot, where observing spectral similarities was difficult due to strong mixing between species and samples (Figure 5b). Data exploration through PCA disclosed that spectral samples of different tree species were heavily mixed, that is, similar to one another. There were no clear patterns observable when visually inspecting the datapoints and clusters. This also indicates that the required information for complete tree species separability after PCA transformation is not entirely held within the portion of variance captured by the first three PCs. Nevertheless, because the first two PCs explained close to 100% of variation, PCA can be useful for dimensionality reduction of tree bark spectra (VIS to NIR), while keeping low approximation error of the data present. We inspected the first three PCs and their loadings (Figure A1), and noticed that PC1 represents an equally weighted sum of the wavelengths in VIS and an equally weighted sum of reflectance in NIR (both positive). The coefficient loadings in VIS were about half of the loadings in NIR, so PC1 seems to explain the average or general reflectance of the bark samples. The second PC offers discrimination between the VIS and NIR wavelengths, that is, positive loadings in VIS and negative loadings in NIR. Despite almost a negligible percentage of the total variance explained, the third PC is a weighted contrast of the red‐edge region (negative loadings), to the adjacent VIS and NIR wavelengths (both with positive loadings).

### 3.3 Linear discriminant analysis

**FIGURE 6:**
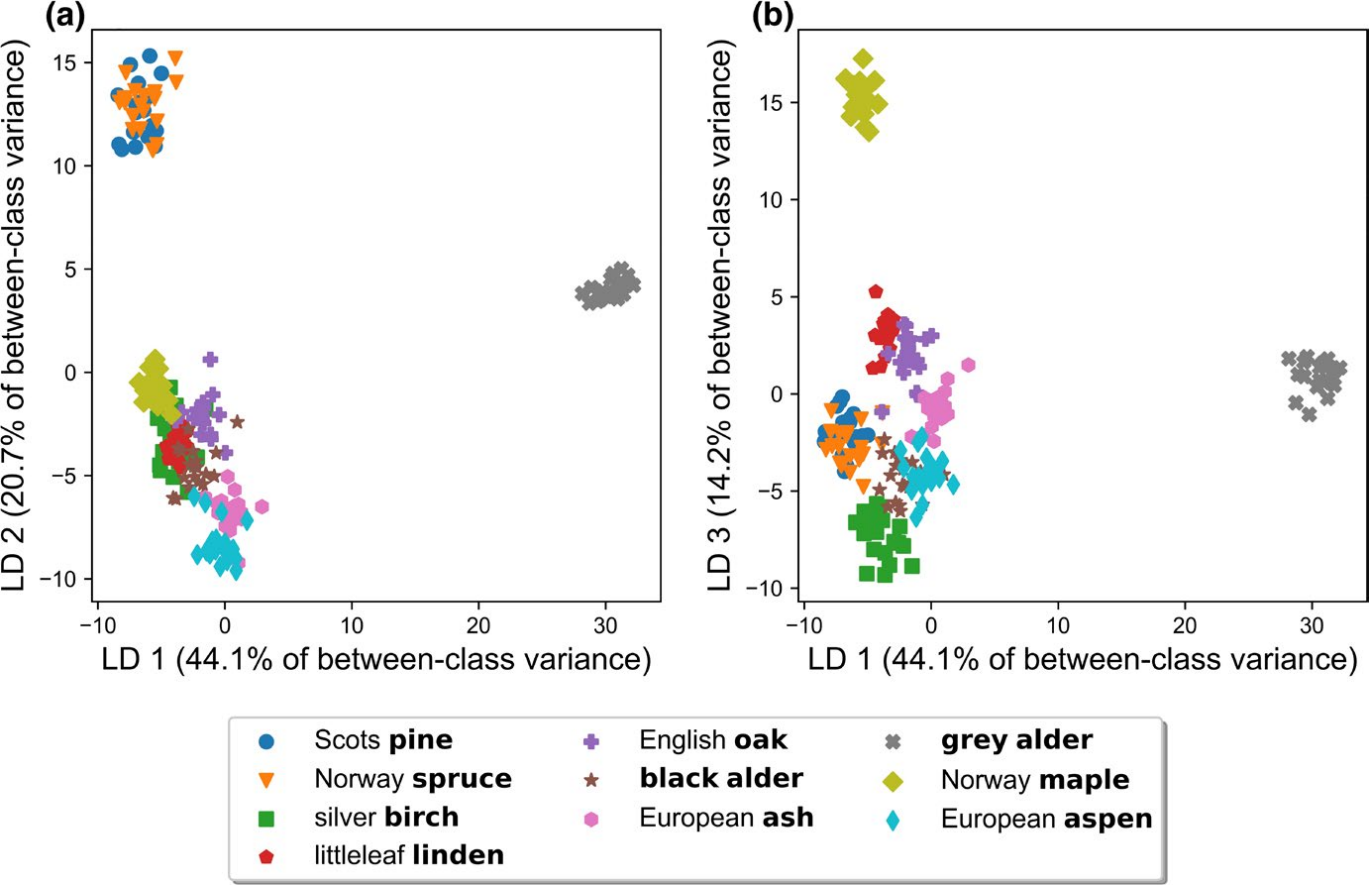
Two‐dimensional projections of bark reflectance samples into linear discriminants (a) one and two (LD1 and LD2), and (b) one and three (LD1 and LD3). Axis labels show the percentage of explained between‐class variance by each discriminant

**FIGURE A2:**
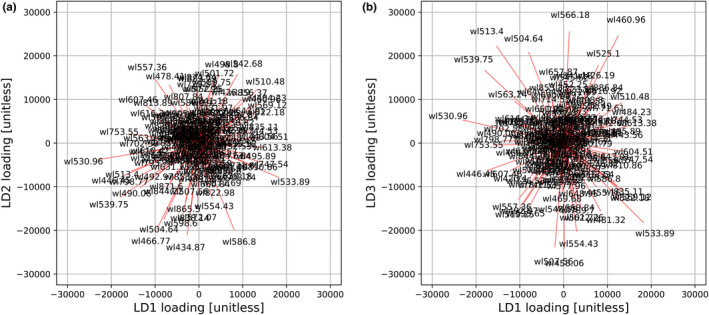
The loadings for the linear discriminants (a) one and two as vectors (LD1 vs. LD2), and (b) one and three as vectors (LD1 vs. LD3). The red lines are the vectors and the black text with prefix “wl” at the end of each vector gives the wavelength it is connected to. See Figure A3 for a different visualization of the loadings for each linear discriminant (LD1–3) separately

**FIGURE A3:**
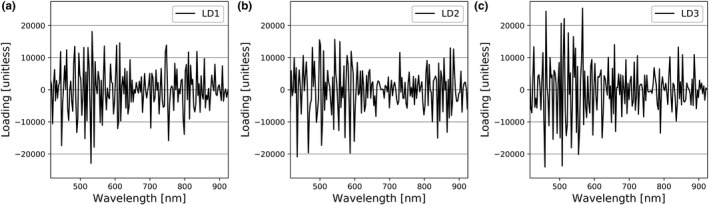
The loadings of the linear discriminants (a) one (LD1), (b) two (LD2), and (c) three (LD3) plotted against wavelengths

Corresponding with PCA in section 3.2.1, we present the visualization of the first three discriminants (LD1–3) from the LDA (Figure 6, Figures A2 and A3). The first three LDs (from total of nine) captured 79% of the between‐class variance in the data. In detail, LDs 1–3 retained 44.1%, 20.7%, and 14.2% of the between‐class variance, in ascending order. LDA produced projections of the input data that highlighted the differences between tree species more clearly than PCA (Figure 5 vs. Figure 6). The more tightly clustered samples made the comparison of distances between species visually easier, as the overlaps between species were kept to a minimum. The input data projected onto the first and second discriminants (LD1 and LD2) produced a cluster of gray alder samples that was clearly separated from the other species by the first LD (Figure 6a). The two coniferous species spruce and pine were also located separately from the deciduous species, in the opposite extremes of LD1 and LD2. The clusters produced by spruce and pine samples were heavily mixed with each other (Figure 6a). The seven remaining tree species of ash, aspen, birch, black alder, linden, maple, and oak formed tight clusters within species, but they also formed a relatively dense and mixed species cluster (Figure 6a). When projecting the data to the first and third LD (Figure 6b), gray alder and maple formed their own distinct clusters that were separated clearly from the other species (Figure 6b). Interestingly, oak and linden, spruce and pine, aspen and black alder were clustered closely as pairs in this projection of LD1 and LD3 (Figure 6b). Ash and birch remained close to the described three pairs, but more separately on their own (Figure 6b). From the two visualizations in Figure 6, and from the lesser LDs viewed, but not shown here, we discovered four clusters: (1) gray alder, (2) maple, (3) spruce and pine, and (4) a large group consisting of species that were not clearly separated.

### 3.4 Taxonomic relatedness and hierarchical clustering

**FIGURE 7:**
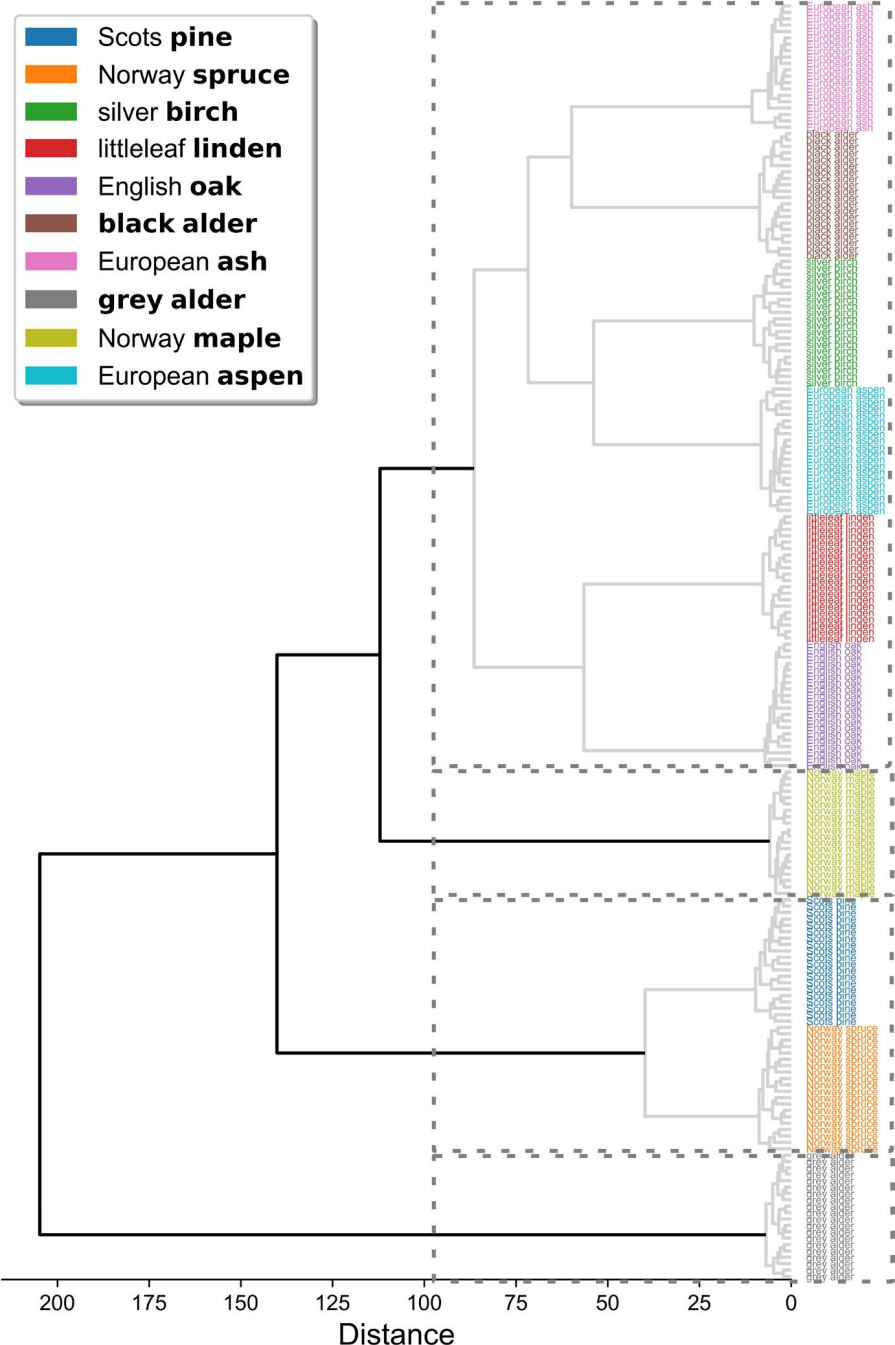
Dendrogram visualizing the results of the agglomerative clustering of tree species based on linear discriminants (outputs of linear discriminant analysis) derived from reflectance spectra of stem bark. The gray dashed lined rectangles display four clusters of measured species and samples distinguished by linear discriminants from the measured species’ and samples’ reflectance (see Figure 6)

Because LDA was able to separate species better than PCA, we used the outputs from LDA as inputs for agglomerative clustering analysis. If ten clusters were chosen (i.e., same number as the measured tree species), the dendrogram illustrates clear species‐specific branches, with no samples mixing with another species (Figure 7). On the other hand, if four clusters were chosen based on what we deduced from LDA, then (1) gray alder and (2) maple formed their own individual branches, (3) coniferous spruce and pine formed a branch together, and finally (4) ash, aspen, birch, black alder, linden, and oak clustered in one large branch (the four clusters are highlighted with gray dashed lined rectangles in Figure 7).

To examine whether the spectral properties of our study species could be linked to their taxonomic hierarchy, we compared the spectral similarity of our study species (Figure 7) to their general phylogenetic lineages (Schoch et al., 2020). We used the Taxonomy Common Tree tool (NCBI, 2022) within the database to generate a taxonomic tree (taxonomic labels at various levels of the hierarchy) with the names of our species. We observed that the spectral similarity (based on the dendrogram in Figure 7) was not clearly connected to the corresponding general phylogenetic lineages. Our study species belonged to one of the two classes listed: Pinopsida (containing pine and spruce) and Magnoliopsida (containing the rest of the species). Pine and spruce formed a distinct cluster, but one species (gray alder) formed a separate cluster, which was not grouped with other species under the clades that could describe the classes of Magnoliopsida and Pinopsida. If we exclude gray alder, the two classes could be identified based on stem bark spectra. Overall, taxonomic relatedness based on stem bark spectra was not clear at lower than class level (Table 1).

### 3.5 Reflectance distribution and histogram analysis

**FIGURE 8:**
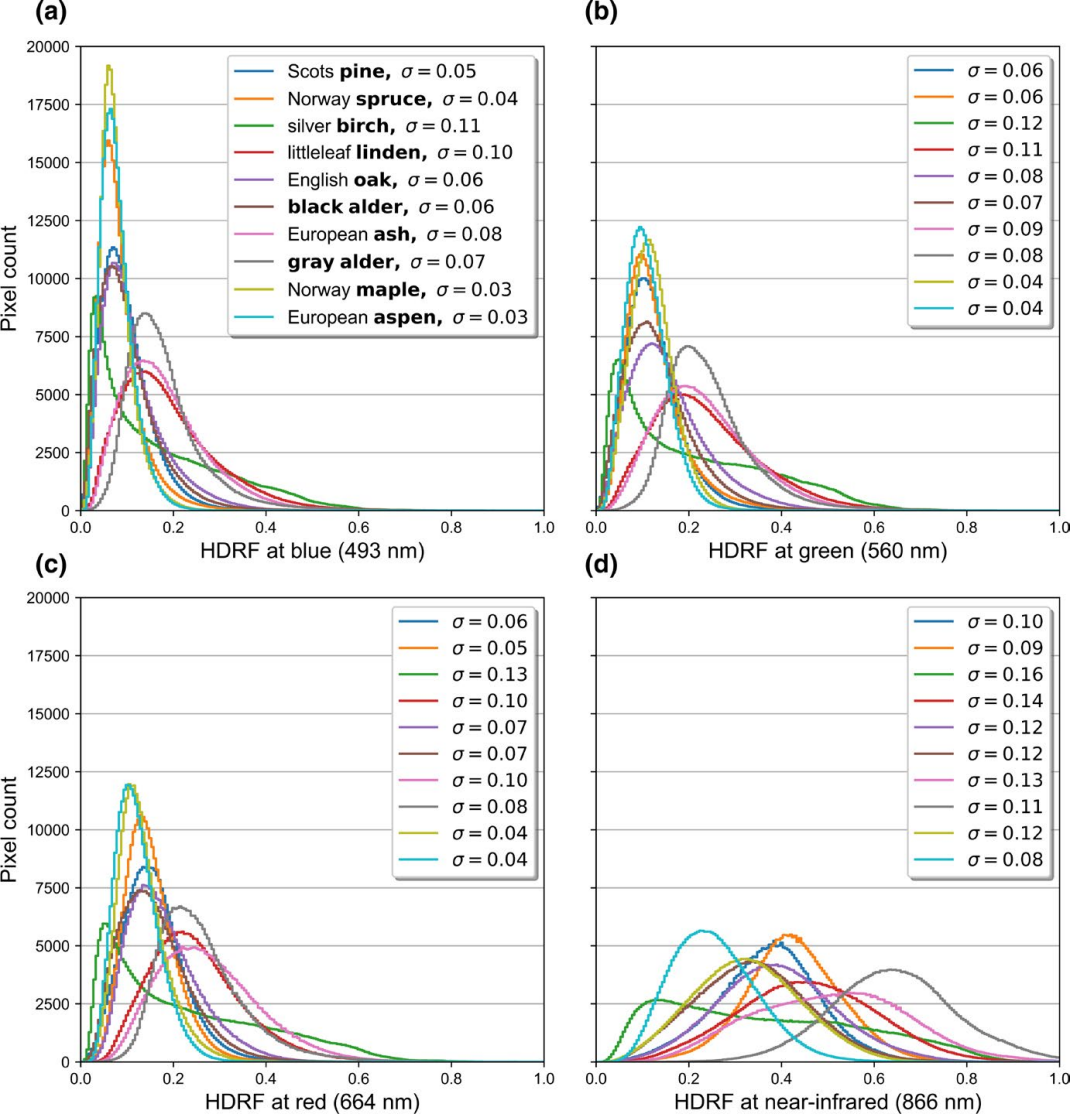
Average species‐specific reflectance histograms for four different wavelengths: (a) blue at 493 nm, (b) green at 560 nm, (c) red at 664 nm, and (d) near‐infrared at 866 nm. Sigmas (σ) next to the names of tree species are the mean standard deviations, calculated from using all pixels from all samples per species

We analyzed the intraspecific distribution of reflectance values within hyperspectral images with average histograms for blue, green, red, and NIR wavelengths (Figure 8a–d). The histogram analysis supports the earlier results that standard deviations increase from VIS to NIR (Figure 2a). It also demonstrates that birch reflectance does not display normal distribution characteristics (Figure 8a–d) and that even though standard deviations of reflectance values within hyperspectral images might be similar among species, the distributions of reflectance can still be uniquely different (Figure 8a–d).

## 4 DISCUSSION

### 4.1 Intra‐ and interspecific variability of bark spectra

We analyzed the VIS–NIR reflectance spectra of stem bark for ten common boreal and temperate tree species. The spectral signatures were computed from hyperspectral images that were collected in the field utilizing a mobile imaging spectrometer. The results showed that the reflectance spectra of bark have generally similar shapes across VIS–NIR wavelengths, which differ mostly in the level of reflectance. This characteristic shape of bark spectra can be found even from Amazonian tree species measured by Hadlich et al. (2018) and from the outer bark spectra of *Boswellia papyrifera* (Del.) Hochst measured in Ethiopia by Girma et al. (2013). Furthermore, our results indicated that the bark spectra of our study species are similar in VIS but more distinctly diverging in NIR. This suggests that NIR wavelengths are more promising for differentiating tree species spectrally from one another, because the spectra considerably overlap in VIS. Similarity of spectra in VIS between different species was also noted by Campbell and Borden (2005) for conifers and angiosperms measured in western North America. In our study, the interspecific similarity in VIS was present across taxonomic families and two different forest biomes. In contrast, when visually comparing our results to Hadlich et al. (2018), the reflectance spectra of selected Amazonian tree species were generally lower in VIS and slightly higher in NIR. Hence, the general increasing trend in bark spectrum as a function of wavelength is present in all species, but the level of reflectance in VIS–NIR shows slightly different trends from one forest biome to another. Pigmentation, chlorophyll content, surface moisture, carbon constituents, residual moss, and lichen (i.e., epiphytes) (Asner, 1998; Goward et al., 1994; Roberts et al., 2004) have all been suggested to cause interspecific differences in the spectra of bark, but most of these spectra‐biochemical relationships still have not been studied in depth. Finally, seasonal dynamics of bark spectra (due to the presence of chlorophyll in the bark, as, e.g., in aspen) might also affect the interspecific differences, but this has not been studied to our knowledge.

Spectra of four tree species could be compared with previous studies and were noted to have similar shape and level of reflectance: silver birch (Juola et al., 2020; Lang et al., 2002), Norway spruce (Forsström et al., 2021; Juola et al., 2020; Lang et al., 2002; Williams, 1991), Scots pine (Forsström et al., 2021; Juola et al., 2020; Lang et al., 2002), and black alder (Clasen et al., 2015; Kuusk et al., 2013). Notably, VIS–NIR stem bark spectra of littleleaf linden, English oak, European ash, gray alder, Norway maple, and European aspen have not been previously published. Mean spectra of our study species showed a similar transitional change in the red‐edge region as noted by Hall, Huemmrich, Strebel, et al. (1992). Standard deviations and coefficients of variation of spectra are in line with previous studies (Asner, 1998; Campbell & Borden, 2005; Goward et al., 1994; Hall, Huemmrich, Strebel, et al., 1992; Juola et al., 2020; Kuusk et al., 2013; Lang et al., 2002; Roberts et al., 2004), and thus confirmed the findings that stem bark spectra are highly variable. Tree species with characteristic stem bark, such as birch, stood out clearly when inspecting variation within the data. Excluding birch, the intraspecific variation was similar between our study species and was relatively stable from VIS to NIR wavelengths.

Asner (1998) reported that coefficients of variation for bark spectra were the highest in NIR, whereas we found them to be higher in VIS rather than NIR. Goward et al. (1994) reported that bark spectra from their broadleaf species were two to three times more reflective than that from their conifer species, while our study species showed much smaller differences between the two. Roberts et al. (2004) described conifer bark spectra being concave and deciduous broadleaf bark spectra being convex in shape, yet such clear distinctions between species were difficult to observe in our results (i.e., general shape of spectra were similar for all measured species). The methodological difference in our results, when compared to previous studies, is that we present bark spectra that have been derived from hyperspectral images and not from non‐imaging point spectrometers. Measuring small bark samples that have been detached from the tree, as has often been previously done, includes the risk of altering the natural state and spectrum of stem bark.

Finally, we utilized the data to obtain information on the distributions of reflectance values within the samples for each species. Histogram analysis highlighted that the mean distributions were species‐specific. Consequently, the distributions of reflectance values within hyperspectral images can potentially be used to retrieve information about the differences between species that is not available in the mean spectra of samples alone. Thus, a mobile imaging spectrometer setup is a good option for acquiring spectroscopic information of stem bark for tree species in different biomes and remote forests.

### 4.2 Taxonomic relatedness based on reflectance of bark

Two different dimensionality reduction methods (PCA and LDA) were applied on our data to reveal possible species clustering of spectral samples. Application of PCA did not separate the spectral samples of our study species into clear clusters. This was an interesting result that supported the fact that stem bark reflectance spectra are very similar between samples of our study species in the VIS–NIR region. With PCA, we also found that the bark spectra in VIS–NIR could be reduced to 3 or 3 dimensions, from the original input of 173 wavelengths, without losing much information. Hence, PCA was able to efficiently compress and preserve much of the information contained in the spectral signatures. On the other hand, LDA succeeded in maximizing the differences between groups of species, resulting in the species clusters being visually more distinguishable from each other. This also means that known class labels (i.e., information on species) were required to gain best separability between the clusters of species. This supports the argument that the stem bark of boreal and temperate tree species is spectrally similar in VIS–NIR wavelengths.

In this study, we could distinguish class and higher clade levels using VIS–NIR hyperspectral imaging spectroscopy of stem bark samples. In comparison, with Fourier transform NIR (FT‐NIR) spectroscopy and absorbance spectra of branch bark and leaves of Amazonian trees, Lang et al. (2017) obtained good discrimination on species, genus, and family levels. On the other hand, Cavender‐Bares et al. (2016) discovered with VIS to shortwave‐infrared (SWIR) reflectance spectra of leaves (obtained with a full range VIS–SWIR point spectroradiometer) that there is potential for better taxonomic classification when going from species level to higher hierarchical levels. The range of possibilities that optical spectroscopy technologies could provide for biodiversity studies and field surveys remains open for future research and novel implementations.

## 5 CONCLUSIONS

Our study highlighted the intra‐ and interspecific variation of bark reflectance of boreal and temperate tree species. The spectral library collected in this study contributes toward building a comprehensive understanding of the spectral diversity of forests needed not only in remote sensing applications but also in, for example, biodiversity or land surface modeling studies.

## CONFLICT OF INTEREST

The authors declare there are no conflicts of interest.

## AUTHOR CONTRIBUTIONS

**Jussi Juola:** Conceptualization (equal); Data curation (lead); Formal analysis (lead); Investigation (lead); Methodology (equal); Visualization (lead); Writing – original draft (lead). **Aarne Hovi:** Conceptualization (equal); Formal analysis (equal); Investigation (equal); Methodology (equal); Supervision (equal); Writing – review & editing (equal). **Miina Rautiainen:** Conceptualization (equal); Funding acquisition (lead); Investigation (equal); Project administration (lead); Resources (lead); Supervision (lead); Writing – review & editing (equal).

## Data Availability

Juola, Jussi; Hovi, Aarne; Rautiainen, Miina (2022), “A dataset of stem bark reflectance spectra for boreal and temperate tree species”, Mendeley Data, V2, https://doi.org/10.17632/pwfxgzz5fj.2
